# FAITH-BASED HOME ACTIVITY TOOLBOX FOR BLACK FAMILIES AFFECTED BY DEMENTIA

**DOI:** 10.1093/geroni/igad104.1268

**Published:** 2023-12-21

**Authors:** Fayron Epps, Katie Pierson, Mayra Sainz, Janelle Gore

**Affiliations:** Emory University, Atlanta, Georgia, United States; Emory University, Atlanta, Georgia, United States; Emory University, Atlanta, Georgia, United States; Centers for Disease Control and Prevention, Atlanta, Georgia, United States

## Abstract

The purpose of this study is to explore the effectiveness of a culturally relevant and faith-based home toolbox, Faith-HAT, for Black caregivers of people living with dementia (PLWD). Caregivers face high levels of stress, anxiety, caregiver burden, and depression, and engaging in religious activities often serves as a protective factor to promote better mental health. Despite the significant role of religiosity among caregivers, few resources exist for religious Black caregivers. A toolbox was developed to improve the physiological health of Black caregivers and provide caregivers and PLWD with meaningful activities to engage in. Participants were recruited from the Atlanta Metropolitan Area (n=16). Caregivers all cared for a family or friend experiencing moderate to severe dementia and had a history of participating in religious activities. Over 6 weeks, the caregiver and PWLD engaged in Faith-HAT, selecting different activities to complete three times a week. Journal entries were made a total of 12 times (at the beginning and end of each week), and caregivers met online with a research team member for weekly updates and baseline, midline, and end-line interviews. Preliminary results suggest engaging with the toolbox has reconnected the caregiver and PLWD, cultivated religious connectedness, and supported continuation of caregiving activities. This faith-based, family-oriented, person-centered approach is geared towards enhancing the quality of life for families affected by dementia and draws on powerful Christian principles from within the black community to achieve this goal.

